# Identification of hidden health utilization services and costs in adults awaiting tertiary care following mild traumatic brain injury in Toronto, Ontario, Canada

**DOI:** 10.2217/cnc-2016-0009

**Published:** 2016-08-08

**Authors:** Cindy Hunt, Katrina Zanetti, Brian Kirkham, Alicja Michalak, Cheryl Masanic, Chantal Vaidyanath, Shree Bhalerao, Michael D Cusimano, Andrew Baker, Donna Ouchterlony

**Affiliations:** 1 Head Injury Clinic, St. Michael's Hospital, Toronto, ON, Canada; 2 Dalla Lana School of Public Health, University of Toronto, Toronto, ON, Canada; 3 Brain & Spinal Cord Program & MSK Program, Toronto Rehabilitation Institute, Toronto, ON, Canada; 4 St. Michael's Hospital, Toronto, ON, Canada; 5 University of Toronto, Toronto, ON, Canada; 6 Department of Critical Care, St. Michael's Hospital, Toronto, ON, Canada; 7 Department of Anesthesia & Surgery, University of Toronto, Toronto, ON, Canada; 8 Faculty of Medicine, University of Toronto, Toronto, ON, Canada

**Keywords:** concussion, economic burden of disease, healthcare utilization, mild traumatic brain injury, traumatic brain injury

## Abstract

**Aim:**

The cognitive, emotional, behavioral and physical impairments experienced by adults after mild traumatic brain injury (mTBI) can produce substantial disability, with 15–20% requiring referral to tertiary care (TC) for persistent symptoms.

**Methods:**

A convenience sample of 201 adult patients referred to TC as a result of mTBI was studied. Self-reported data were collected at first TC visit, on average 10 months postinjury. Patients reported the type and intensity of healthcare provider visit(s) undertaken while awaiting TC.

**Results:**

On average males reported 37 and females 30 healthcare provider visits, resulting in over $500,000 Canadian dollars spent on potentially excess mTBI care over 1 year.

**Discussion:**

Based on conservative estimate of 15% of mTBI patients receiving TC, this finding identifies a possible excess in care of $110 million for Ontario. Accurate diagnosis of mTBI and early coordination of follow-up care for those needing TC could increase cost–effectiveness.

In North America, traumatic brain injury (TBI) is a leading public health problem for adults [1]. Mild TBI (mTBI) accounts for 70 to 90% of all TBI cases [2,3]. The incidence of mild TBI in Ontario Canada has increased threefold in the past 4 years to a current estimate of 1800/100,000 annually for adults 18 years and older [4]. Most patients recover within 3 months, yet 15–20% of patients experience persistent symptoms affecting their ability to return to school, employment and regular life activities. Adults with persistent symptoms are usually referred to tertiary care (TC) clinics [5]. In Ontario current wait times for TC are lengthy and average 7 months [3]. Patients are essentially on their own to endure the protracted wait times.

Much of the existing literature on service utilization following a TBI looks at patients who were hospitalized following their injury [5–7]. There has been relatively little research examining patterns of health service and costs or factors associated with variation in costs following mTBI [8]. In Ontario, the majority of adult mTBI cases are not hospitalized. As a result data specifically referencing patient's healthcare utilization patterns post mTBI is unreported, and the respective cost burden is elusive.

The aim of the current study was to describe the type(s), intensity and estimate costs of healthcare provider (HCP) visits in a sample of adult patients referred to TC following an mTBI in Toronto, Canada. Toronto is the provincial capital of Ontario and ethnically diverse. The metropolitan population is over 6 million making Toronto the largest city in Canada.

## Methods

### Study population & study instrument

The study population was comprised of 335 mTBI patients who were referred by a clinician to be seen for their first appointment at a TC outpatient head injury clinic between June 2013 and May 2014. All information collected from patients was clinical data with aggregate analysis approved by the institutional review board of the hospital. Descriptive statistics were performed to characterize the demographic and TBI-related factors of the population: age, gender, time since injury and injury-causing activity.

Patients were given a clinical questionnaire to complete during their first visit to the TC. Self reported information included demographic and injury characteristics, current health status, quality of life, employment and determinants of health. The questionnaire also asked which health services patients accessed since their recent head injury, and the number of visits to each of the HCP(s) type(s), specifying that only visits relevant to the most recent head injury should be recorded. No existing standardized question about healthcare utilization could be located. The question developed read: “Indicate the number of times you have visited or accessed the following health services to support you with your most recent head injury (the head injury that brings you to the clinic today). Choose all that apply”. Participants checked off the following HCPs that were applicable to them and indicated the “number of visits since most recent head injury” for each: Emergency department, family doctor, walk-in clinic, psychiatrist, neurologist, ENT (ear, nose and throat) doctor, physiotherapist, occupational therapist, psychologist, social worker/case worker, massage therapist, home healthcare, osteopath, acupuncturist, chiropractor, other, and “I have not used any of these services”.

In addition, the presence of comorbid health conditions was determined in the clinical questionnaire. This question asked: “Have you been diagnosed with any of the following?” In checkbox format, participants checked off the following conditions that were applicable to them: none, learning disability, attention deficit disorder/hyperactivity disorder, depression, anxiety, other mental illness, sleep disorder, migraine headaches, chronic fatigue syndrome, fibromyalgia, visual disturbances, epilepsy/seizures, chronic illness (i.e., heart disease, respiratory disease, diabetes, cancer), infectious disease (i.e., HIV, AIDS, TB), and other.

### Health service utilization

Descriptive statistics were used to summarize data for the types of health services accessed by patients and the intensity at which they were used. Responses were excluded when patients reported the frequency of service utilization non-numerically (i.e., ‘many times,’ ‘lost count,’ ‘a few,’ etc.). Intensity of service use per patient was compared between age groups to determine whether any age group was at higher or lower risk of having high utilization of health services. This was conducted using a nonparametric Kruskal–Wallis one-way analysis of variance. To compare the per-patient intensity of health service use between males and females, a Shapiro–Wilk test was first used to test for normality, followed by a Mann–Whitney U test, a nonparametric alternative to a t-test.

Shapiro–Wilk tests of normality followed by Mann–Whitney U tests were conducted to compare the number of total health service visits of patients who did and did not report being previously diagnosed with each health conditions. A significance level of p < 0.05 was used for all statistical tests. All statistical analyses were completed using the computer software IBM SPSS Statistics (Version 20).

### Cost calculation

The cost per visit to an HCP was obtained from the 2014 Ontario Health Insurance Plan (OHIP) Schedule of Benefits for OHIP-covered services [9]. For services not covered by OHIP, cost per visit was obtained from the Professional Services Guideline [10]. Wherever different fees were found for different visit types to the same HCP (e.g., consultation fee differing from follow-up visit fee), the fee for a consultation visit was used. The data analyzed in this study did not allow us to determine which type of visit patients used (i.e., multiple visits to the same HCP reported by a patient could have been for one initial consultation visit and the remainder consisting of follow-up visits, or multiple consultation visits to multiple physicians of the same HCP type). Where applicable, fees for 1 h of the service were used.

The cost calculation did not include any indirect costs incurred by the patient or patient's family, such as loss of income as a result of the injury. Reported visits to other HCP types not itemized in the questionnaire were not quantified, due to significant variability in cost per visit among different HCPs.

## Results

### Participant demographics & characteristics

During the study period, 335 patients were seen for their first appointment in the head injury clinic and were administered a questionnaire. However, 201 responses were received with complete data to the question pertaining to health service access, and only data from these 60% (201/335) were analyzed in this study. Of the 201 patients who responded to the question about health service access, mean age was 42 years (range 16–90; standard deviation [SD] 16.8). There were slightly more males (55%) than females (45%). The mean length of time since injury at the time of assessment was 10 months (range 2 days-7 years; SD 13 months). Almost half (42%) of patients sustained their head injury by some transportation-related means, followed by 21% of patients sustaining their injury as a result of a fall (Table 1).

**Table 1.** **Demographics and injury characteristics.**

**Demographic characteristic**	**Response**
Age	42.2 ± 17.4 years (range 16–90)

Gender	55% (n = 111) male 45% (n = 90) female

Time since injury	Mean 10 ± 13 months postinjury (range 2 days–7 years)

Injury-causing activity	51% transportation 21% falls 13% sports 8% violence 1% blast 6% unknown activity

### Health service utilization

**Figure 1.**
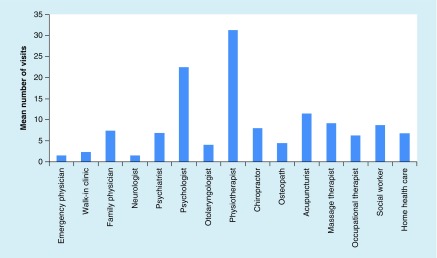
**Mean per-patient number of visits to each healthcare provider between time of injury and first tertiary care head injury clinic visit (service intensity).**

The total number of HCP visits was 6794 for the 201 participants. The mean number of healthcare visits to each provider per patient is shown in Figure 1. The data are presented by HCP type, among those who accessed the service at least once, measuring service intensity. The highest mean number of visits was for physiotherapy (31 visits), presumably because individuals who utilize physiotherapy services do so on a consistent basis, and likely recorded an estimate of the total number of received visits to date. The HCP with the second highest mean number of visits were psychologists (22 visits), followed by acupuncturists (11 visits).

Shapiro–Wilk test of normality rejected the null hypotheses that the frequencies of all health service visits for males and for females were distributed normally (p < 10–6). Males had an average of 37 visits to an HCP between injury and their first appointment in the clinic, while females had an average of 30 HCP visits. However, this difference was insignificant (U = 4931, p = 0.876). Mann–Whitney U test also revealed no significant difference in the length of time since injury between males and females (U = 3895.5, p = 0.774).

The total number of healthcare visits was not significantly different between any age groups examined (≤25, 26–35, 36–45, 46–55, 56–65, 66+ years) (χ^2^(5) = 8.744, p = 0.261). Similarly, no statistically significant differences were found in the total number of visits to an HCP between patients who reported being diagnosed with any comorbid health conditions listed in section 2.2 and patients who did not.

### Economic cost

Costs of health service visits to some HCP types in this analysis (emergency department, walk-in clinic, family physician, neurologist, psychiatrist and otolaryngologist) were calculated from the government payer's perspective, as Ontario has a public healthcare insurance program and these services are covered by OHIP. The remainder of the services were reported from the individual patient or family perspective, as these health services are generally not covered by OHIP, but can be covered in certain circumstances. These services may require out-of-pocket or private insurance coverage by the patient. Table 2 shows the total estimated costs for all health service visits of the whole study cohort by HCP type. Costs are reported in 2014 Canadian dollars.

**Table 2.** **Total costs for health service visits in study cohort.**

**Healthcare provider type**	**Total cost for cohort (Canadian dollars)**
Emergency department	19,227.20

Walk-in clinic	3396.80

Family physician	72,568.00

Neurologist	15,871.50

Psychiatrist	34,695.60

Psychologist	65,984.60

Otolaryngologist	701.10

Physiotherapist	169,643.76

Chiropractor	75,912.20

Osteopath	7245.00

Acupuncturist	41,300.00

Massage therapist	31,141.80

Occupational therapist	28,867.12

Social worker/case worker	11,822.35

Home healthcare	6886.36

Total	585,263.39

The highest cost ($169,643.76) was incurred for visits to physiotherapists and the lowest total cost was for otolaryngologist visits ($701.10). The aggregate total cost of all 6794 visits for the whole cohort was approximately $585,263.39.

## Discussion

The aim of the current study was to describe type(s), intensity and estimate costs of HCP visit(s) during the interval postinjury and prior to TC in a convenience sample of patients experiencing a mTBI and who were referred by a clinician to TC. On average there was a 10-month wait time prior to their TC appointment. Upon their first TC appointment patients were asked to recall the number of HCP visits they received between time of injury and their first TC appointment. Data were extracted from charts for analysis. In the study sample of 201 mTBI patients a total of 6794 HCP visits which could be considered ‘excess’ care. In aggregate this resulted in an estimated total direct cost of $585,263.39. Based on the Ontario public healthcare insurance payments the total cost accrued for these additional HCP visits reached $3000 per patient. Overall these findings are consistent with other studies that found mTBI patient care can accrue high costs and were as likely as those with severe TBI to use a variety of services postinjury [8,11].

Placing this estimate in context with the current Ontario population of 14,000,000 and adult incidence of mTBI in Ontario of 1800/100,000 as well as the (conservative) incidence of 15% of mTBI patients with persistent symptoms referred to TC, results in an outstanding previously unreported heath service cost of over $11 million dollars annually in Ontario. This estimate could be considered conservative, as it did not take into account indirect costs such as lost income, transportation to and from appointments and intangible costs associated with diminished quality of life. In addition, total costs of consultation visits from the OHIP Schedule of Benefits were taken for cost calculation purposes, however procedures (diagnostic tests or imaging) or medications could have also been ordered by the physician, or the visit could have been coded as a type other than a consultation visit, potentially with a higher associated fee.

The HCP services with the highest average number of visits included: physiotherapists, psychologists and acupuncturists. This differed somewhat from previous studies examining health service utilization post-TBI reporting services most used postinjury were specialist physicians, general practitioners, physiotherapists and occupational therapists [5,12]. No significant differences were found between the number of total HCP visits by age or sex. Although Ontario has a universal healthcare system, most community rehabilitation services are privately funded, leaving patients and families responsible for payment unless benefits are available from third party sources. Often even the third party payments have an expiration date (i.e., 3 months postinjury) which begs a future research question as to who is paying for the ‘excess’ cost of care.

Few research studies have investigated health service utilization patterns in patients after a TBI Ontario that are not related to hospitalization. This study demonstrates that understanding patterns of healthcare service utilization is critical for planning and optimizing available resources to adults who have sustained an mTBI, particularly those that need TC [13].

Patients who are not hospitalized are essentially on their own to endure the protracted wait times when referred to TC. The significant number of visits and cost found by this study highlight the importance of appropriate and timely mTBI management. Increased accuracy of diagnosing mTBI is needed, as this may help to allay the high costs incurred by this population. Ryu *et al*. [14] found a 43% rate of disagreement in diagnosis of mTBI in family physician and emergency department settings when a retrospective chart review was undertaken by a head injury expert. Directing diagnosed mTBI patients to the most appropriate course of follow-up care after assessment in primary care settings is essential. These efforts could decrease costs through eliminating unnecessary visits, such as returning to the emergency department. Importantly, the large number of health service visits also reflects the difficulty that patients experience in trying to find support for their symptoms while they wait for TC.

Utilization of health services and actual healthcare spending occurs disproportionately among a small ‘high-cost users’ subset of the population [11]. Two thirds of total healthcare expenditures for Ontario were accounted for by the top 5% of healthcare users, while just 1% of the expenditures were spent on the bottom 50% [15]. Further research has demonstrated an important link between socioeconomic status and risk of being high-cost users of the healthcare system [11]. As improving outpatient management for high-cost patients has been identified as a priority within healthcare [16], it would be helpful to identify characteristics of TBI survivors who are high-intensity users of HCPs and their health outcomes compared with lower-intensity users with similar injury and socio-economic characteristics. The potential for linking administrative data together with the data tabulated in this study should also be explored, as this would provide insights into the characteristics of high-intensity users for this patient population. Future work involving administrative data could also allow for seeing whether confounders, such as preexisting health conditions or other injuries, may be responsible for some of the visits. Such research could have important implications for policy and resource planning.

Future analysis could compare the use of healthcare services and costs between younger and older patients following mTBI. Future work involving control groups would increase the accuracy of the estimate of the number of visits and costs that can be attributed to the mTBI itself, rather than some visits being in support of other health conditions. Ideally, future research could ask the same questions to control groups of patients: a group with orthopedic injuries as well as a control group of uninjured healthy individuals, matched with an mTBI group for comorbidities. This would provide insight into the proportion of visits and costs reported in the present study that may have been in support of another health condition rather than the mTBI. Finally, further study of service utilization could include a cohort of mTBI patients collecting data at multiple points over time after being seen in a head injury clinic, exploring trends in type and intensity of service utilization over time.

### Limitations

The degree to which this study population is representative of all mTBI patients, who are referred to TC is not definitively known. The participant group may have been biased toward more significant injuries and comorbidities that translate into higher healthcare utilization patterns [17]. Another limitation surrounds the lack of information on patient outcomes. What is deemed ‘excess’ care after injury and before the first TC visit is difficult to ascertain without information about clinical outcomes. Furthermore, the wide age range in this study (16–90 years) could be masking important heterogeneity across the life span supporting the need for age specific healthcare services.

This research has limited generalizability across geographical regions of Canada, as the reported costs are for Ontario. Fees per visit by services are controlled by provincial government health insurance rates. The observational study design does not allow for determining if the mTBI alone has a causal effect on HCP visits and cost. Many other confounders that were not examined may have contributed to study limitations such as co-morbidities. Recall is often compromised in the TBI population. The fact that patients may have had difficulty remembering and recounting quantities of health service visits is a limitation of the self-report method employed.

### Conclusion

This study identifies previously unreported patterns of HCP visits in Ontario to adult patients following mTBI who were referred to TC. In this modest cohort of 201 mTBI patients referred to TC, over $500,000 Canadian dollars was spent on 6794 pre-TC HCP visits, while patients waited on average 10 months for their first TC appointment. These findings give new insight into the challenges of mTBI care and in understanding of the true burden of TBI occurring each year in Ontario. This information can be useful to clinicians and policy makers, advocating for early identification of mTBI, in particular those with risk factors for persistent symptoms by further highlighting the need of timely treatment.

**Executive summary**

- Health service utilization and associated costs for patients with mild traumatic brain injury (mTBI) awaiting tertiary care (TC) warrants further investigation.
- We conducted a retrospective chart review of 201 patients suffering from mTBI and identified a sum total of 6794 healthcare provider visits during the average 10-month wait for patients between time of injury and the time of their first TC Head Injury Clinic visit in Toronto, Ontario, Canada.
- Based on the Ontario public healthcare insurance payments the total cost accrued for these additional provider visits reached $3000 per patient/10 months (average).
- Given the current Ontario population of 14,000,000, the incidence of mTBI in Ontario of 1800/100,000 and the conservative incidence of 15% of mTBI patients with persistent symptoms referred to TC; these findings estimate a previously unreported/hidden heath costs of over $110 million annually in Ontario.
